# Comprehensive Survey of Genetic Diversity in Chloroplast Genomes and 45S nrDNAs within *Panax ginseng* Species

**DOI:** 10.1371/journal.pone.0117159

**Published:** 2015-06-10

**Authors:** Kyunghee Kim, Sang-Choon Lee, Junki Lee, Hyun Oh Lee, Ho Jun Joh, Nam-Hoon Kim, Hyun-Seung Park, Tae-Jin Yang

**Affiliations:** 1 Department of Plant Science, Plant Genomics and Breeding Institute, and Research Institute of Agriculture and Life Sciences, College of Agriculture and Life Sciences, Seoul National University, Seoul, 151–921, Republic of Korea; 2 Phyzen Genomics Institute, 501–1, Gwanak Century Tower, Gwanak-gu, Seoul, 151–836, Republic of Korea; Austrian Federal Research Centre for Forests BFW, AUSTRIA

## Abstract

We report complete sequences of chloroplast (cp) genome and 45S nuclear ribosomal DNA (45S nrDNA) for 11 *Panax ginseng* cultivars. We have obtained complete sequences of cp and 45S nrDNA, the representative barcoding target sequences for cytoplasm and nuclear genome, respectively, based on low coverage NGS sequence of each cultivar. The cp genomes sizes ranged from 156,241 to 156,425 bp and the major size variation was derived from differences in copy number of tandem repeats in the *ycf1* gene and in the intergenic regions of *rps16-trnUUG* and *rpl32-trnUAG*. The complete 45S nrDNA unit sequences were 11,091 bp, representing a consensus single transcriptional unit with an intergenic spacer region. Comparative analysis of these sequences as well as those previously reported for three Chinese accessions identified very rare but unique polymorphism in the cp genome within *P*. *ginseng* cultivars. There were 12 intra-species polymorphisms (six SNPs and six InDels) among 14 cultivars. We also identified five SNPs from 45S nrDNA of 11 Korean ginseng cultivars. From the 17 unique informative polymorphic sites, we developed six reliable markers for analysis of ginseng diversity and cultivar authentication.

## Introduction

Korean ginseng (*Panax ginseng* C.A. Meyer), a famous medicinal perennial herb, belongs to the Araliaceae family consisting of about 1,500 species [1]. Although *P*. *ginseng* was domesticated more than 500 years ago, its breeding is difficult due to a long life cycle and low seed yield. In Korea, three local landraces, Jakyung, Chungkyung and Hwangsook, have been cultivated traditionally and nine elite cultivars have been bred and registered through pure line selection from the landraces [2,3]. The nine registered cultivars show many agricultural traits and unique characteristics that are superior to the landraces: Chunpoong is good for red ginseng production; Gopoong contains superior amounts of saponin; Gumpoong is disease-resistant and good for high quality red ginseng production; Sunhyang has a high content of aromatic compounds; Yunpoong, Sunun, and Sunone produce high yields of root; Sunpoong shows excellent root body; and Cheongsun has early germination characteristics [3–5]. Despite this, two local landraces, ‘Jakyung’ and ‘Hwangsook’, are still the main types cultivated in Korea, due to the lack of an established ginseng seed industry.

Chloroplast (cp) genome and 45S nuclear ribosomal DNA (45S nrDNA) sequences are the main molecular targets used for plant taxonomy because these sequences are conserved across plant species and show clear inter-species polymorphism, whereas intra-species polymorphism is rare. Most studies of plant diversity have focused on intergenic spacer (IGS) sequences in the cp genome and on internal transcribed spacer (ITS1 and ITS2) sequences in 45S nrDNA [6–10]. For *Panax* species, we previously identified 60 polymorphic sites at the inter-species level among 101 IGS regions of three *Panax* species, namely *P*. *ginseng*, *P*. *quinquefolius* and *P*. *notoginseng*, using high resolution melting (HRM) analysis [11], but did not find any polymorphism at the intra-species level [12]. In addition, one polymorphism in the 5.8S rRNA region of *P*. *ginseng* cultivars Gumpoong, Gopoong and Hwangsook has been described, and some polymorphisms have been reported among *Panax* species [13,14].

Currently, more than 500 complete cp genomes and a few complete 45S nrDNA sequences have been deposited in GenBank but most species have only a single representative sequence without additional sequence information for related cultivars and/or accessions. Because of this, most studies have aimed to detect genetic diversity at the inter-species rather than intra-species level. Since cp genome and 45 nrDNA sequences are highly conserved within species, only a few studies have reported polymorphism at the intra-species level, including one in onion [15] and one in apple in which cp genome sequences of 47 apple cultivars were used to clarify the domestication history of current apple cultivars [16]. Overall, despite its potential usefulness, the identification and application of intra-species sequence variation has been very limited.

In this study, we generated complete cp genome and nrDNA sequences for nine Korean ginseng cultivars using next generation sequencing (NGS) technology. In addition, we identified 17 polymorphic sites valuable for authentication of ginseng through comparative analysis of those sequences and provide useful markers for authentication of ginseng cultivars and phylogenetic analysis of other *Panax* species and relatives.

## Materials and Methods

### Plant materials

Nine *P*. *ginseng* elite cultivars (Chunpoong (ChP), Yunpoong (YP), Cheongsun (CS), Gopoong (GO), Gumpoong (GU), Sunone (SO), Sunpoong (SP), Sunun (SU), and Sunhyang (SH)) and two local landraces (Jakyung (JK) and Hwangsook (HS)) were used for genomic DNA preparation and sequencing (Table 1). Individual plants (3 ~ 20) of all the cultivars and *P*. *quinquefolius* were used for PCR analysis to validate polymorphic sites. Leaves of mature plants were harvested from the ginseng farm of Seoul National University in Suwon and the Korea Ginseng Corporation (http://www.kgc.or.kr/) and stored at -70°C until use.

**Table 1:** Statistics of WGS and assembly summary for nine *P*. *ginseng* accessions.

Cultivar names	WGS reads for cp assembly			Length (bp) of sequence (GenBank acc. No.)	
	Amounts (Mb)	Genome coverage (x)^a^	Cp coverage (x)^a^	Chloroplast	45S nrDNA^b^
Chunpoong (ChP)	505	0.2	64	156,248 (KM088019)	11,091 (KM036295)
Yunpoong (YP)	1,010	0.3	97	156,355 (KM088020)	11,091 (KM036296)
Gumpoong (GU)	505	0.2	80.00	156,356 (KM067388)	11,067 (KM207667)
Gopoong (GO)	1,010	0.3	325.67	156,355 (KM067387)	10,095 (KM207668)
Sunpoong (SP)	505	0.2	89.04	156,355 (KM067391)	11,012 (KM207671)
Sunone (SO)	1,010	0.3	153.57	156,355 (KM067390)	11,089 (KM207670)
Sunun (SU)	505	0.2	66.73	156,355 (KM067392)	11,025 (KM207672)
Sunhyang (SH)	505	0.2	96.30	156,425 (KM067393)	10,991 (KM207669)
Cheongsun (CS)	505	0.2	99.74	156,356 (KM067386)	10,952 (KM207666)
Hwangsook (HS)	505	0.2	267.90	156,241 (KM067394)	11,070 (KM207673)
Jakyung (JK)	340	0.1	91.19	156,355 (KM067389)	10,964 (KM207674)

^a^ Coverage of genome and cp indicate the total WGS read depth for the complete genome and chloroplast genome, respectively.

^b^ 45S nrDNA length: nearly 1 unit length included full 45S transcription sequence and partial IGS sequence.

### DNA preparation and whole-genome shotgun sequencing

Total genomic DNAs were isolated using the standard cetyltrimethylammonium bromide (CTAB) method [17]. The quantity and quality of genomic DNA were examined using a spectrometer. Whole genomes of nine ginseng cultivars were sequenced using an Illumina genome analyzer (Hiseq2000) by National Instrumentation Center for Environmental Management (NICEM; http://nature.snu.ac.kr/kr.php), Seoul, Korea. Genomic libraries with 300-bp insert size were prepared by following the paired-end standard protocol recommended by the manufacturer and each sample was tagged separately with a different index. Sequencing (101 cycles) was conducted for both ends in a single lane using pooled libraries from nine cultivars. Since P. ginseng cultivars are highly inbred and chloroplasts are maternally inherited, a single specimen of each cultivar and landrace can provide a representative chloroplast type. Therefore, we only sampled one individual plant of each cultivar and landrace for whole-genome shotgun sequencing.

### Cp genome and 45S nrDNA assembly

Assembly of complete cp genome and nrDNA sequences was performed by *de novo* assembly of the low coverage whole genome sequence (WGS) via a bioinformatics pipeline (http://phyzen.com). Briefly, trimmed reads with Phred scores of 20 or less were prepared from the total pair-end (PE) raw reads using the CLC-quality trim tool and then were assembled by a CLC genome assembler (ver. 4.06 beta, CLC Inc, Rarhus, Denmark) with parameters of minimum 200 to 600 bp autonomously controlled overlap size. The principal contigs representing the cp genome were retrieved from the total contigs using MUMmer [18] with the cp genome sequence of *Panax ginseng* cv. ChP (KM088019) as reference sequence. The representative cp contigs were arranged in order based on the previously reported cp genome sequence and connected into a single draft sequence by joining overlapping terminal sequences. Assembly errors were identified in the initial assembly contigs and manually corrected by mapping of raw reads to assembled sequences. Error correction was validated by nucleotide sequencing after PCR amplification.

### Gene annotation

Genes in the cp genome were annotated using the DOGMA program (http://dogma.ccbb.utexas.edu/) [19] and manual curation based on BLAST searches. Circular maps of cp genomes were drawn using OGDRAW (http://ogdraw.mpimp-golm.mpg.de/) [20]. The structures of nrDNA sequences were predicted by comparison with reported ginseng nrDNA sequences, RNAmmer (http://www.cbs.dtu.dk/services/RNAmmer/), and BLAST searches.

### Comparative analysis and development of DNA markers

Cp genome and 45S nrDNA sequences of 11 cultivars were compared with one another as well with using MAFFT (http://mafft.cbrc.jp/alignment/server/) and mVISTA (http://genome.lbl.gov/vista/mvista/submit.shtml).

To validate the intra-species polymorphism in cp and nrDNAs and also to develop DNA markers to authenticate each cultivar, specific primers were designed based on polymorphic sites found in cp genomes and 45S nrDNA among 11 *P*. *ginseng* cultivars. Primers for tandem repeat and InDel regions and derived cleaved amplified polymorphic sequences (dCAPS) primers for SNP sites were designed using dCAPS Finder 2.0 (http://helix.wustl.edu/dcaps/dcaps.html) and the Primer 3 program (http://bioinfo.ut.ee/primer3-0.4.0/), respectively. Genomic DNAs were used as templates for PCR amplification and amplified fragments were analyzed by separation in agarose gels and ethidium bromide staining, as well as by capillary electrophoresis and their separation patterns were analyzed using a Fragment analyzer (Advanced Analytical Technologies Inc., USA) according to manufacturer’s instructions.

## Results

### Complete cp genome and nrDNA sequences of 11 ginseng cultivars

**Fig 1:**
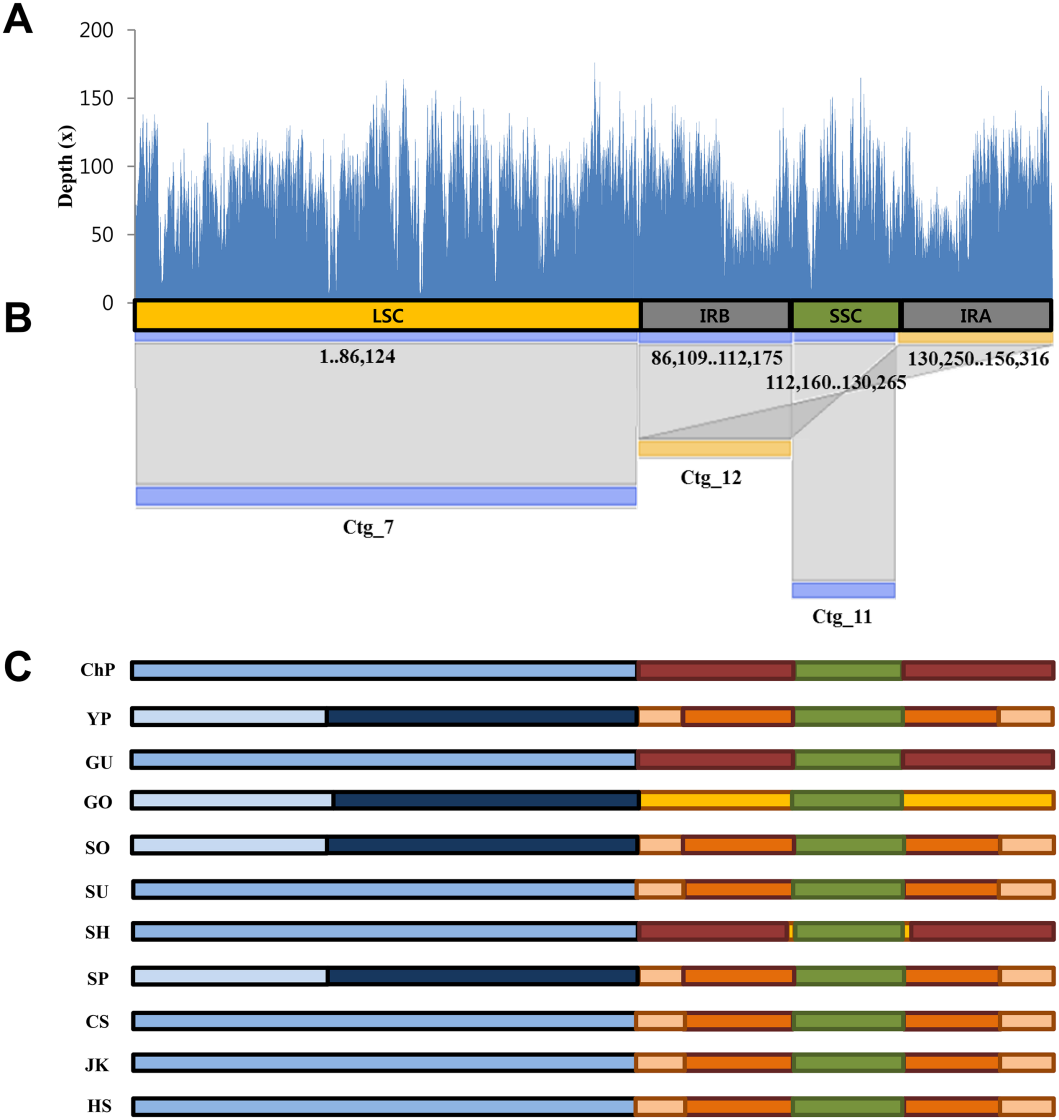
Summary of cp genome assembly of 11 ginseng cultivars. (A,B) Alignment of three contigs covering the complete cp genome sequences of *P*. *ginseng* cv. Gumpoong (GU). (A) Mapping of raw PE reads on the assembled complete GU cp genome. (B) Alignment of the initial contigs representing the cp genome on the complete cp genome sequence. The contig numbers are indicated under the contigs and hit positions are under the reference cp genome sequences for ginseng (ChP cp genome Accession no. KM088019.). The overall structure of the cp genome is denoted with different colored bars: blue, yellow and green, for LSC, IRs, and SSC, respectively. Mapping of 100X raw reads is shown above alignment. (C) Diagram of initial cp contigs used for assembly of complete cp genomes for all 11 *P*. *ginseng* accessions. Different contigs are represented as different colored bars for each cultivar, as detailed in Table 1.

We obtained complete cp genome and nrDNA sequences of each cultivar for 11 ginseng cultivars. We assembled both sequences for each cultivar independently, by *de novo* assembly using low-coverage WGS ranging from 340 ~ 1,000 Mbp, which represents approximately 0.1X ~ 0.3X haploid genome equivalents (Table 1). The entire cp genomes (cp contigs) were obtained by combining three to five contigs for each of the 11 cultivars (Table 1). The cp genome of each cultivar showed coverage of 45.98X ~ 325.67X and the cp genome sequence of the ginseng cultivar Gumpoong is shown as an example (Fig 1A and 1B). In this case, three cp contigs account for the complete cp genome with slight overlap, and exhibit approximately 80X average read mapping depth (Table 1, Fig 1A and 1B). Complete cp genomes for the other ten cultivars were also independently obtained by combining representative contigs (Fig 1C) and manual editing. Complete lengths of the 11 cp genomes ranged between 156,241 bp and 156,425 bp (Table 1). Several sequence assembly errors in the initial contigs were corrected by manual curation and validated by ABI Sanger sequencing.

The 45S nrDNA sequences were each assembled into single contigs. The 45S nrDNA contigs were 10,095~11,089 bp with one gap in the degenerate GC rich repeats in the IGS regions of the various cultivars (Table 1).

### Sequence variations among cp genomes of 14 *P*. *ginseng* accessions

**Fig 2:**
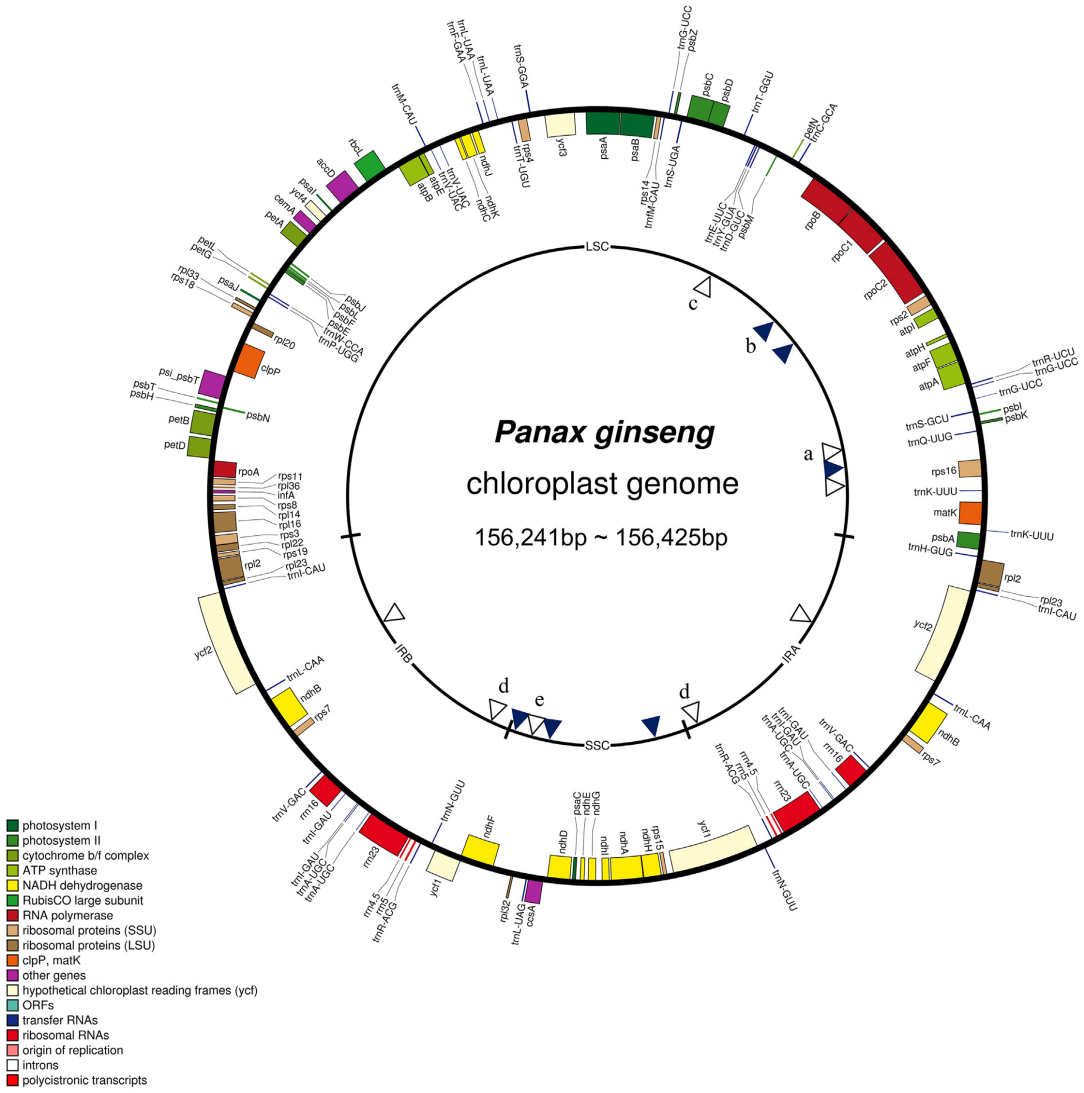
Chloroplast genome map of 11 *P*. *ginseng* cultivars. Colored boxes are conserved chloroplast genes classified based on product function. A total 12 of polymorphic sites among 11 *P*. *ginseng* cultivars are denoted as arrowheads, six white for InDels and six dark blue for SNPs, on the inner circle. PCR markers used in this study (Table 3) are denoted as follows: a, pgcp097f2*r; b, pgcpd01; c, kpcs03; d, pgycf1; e, pgcp139f*r2.

Gene content and order were identical among the 11 cultivars and those previously reported [21] (Fig 2). To investigate sequence divergence in cp genomes among *P*. *ginseng* cultivars, we compared 14 cp genomes accounting for all nine registered elite cultivars and two local landraces in Korea as well as three Chinese ginseng collections. All eleven cultivars are cultivated in Korea. Among them, the nine elite cultivars were bred by pure line selection at Korea Ginseng Corporation (Korea) and are homogeneous, whereas the two landraces, Jakyung and Hwangsook, are admixture-type cultivars cultivated by seed production from farmers. We compared 14 cp genome sequences: the genomes of the 11 cultivars completed in this study (GenBank accession numbers for the cp genomes of the cultivars are displayed in Table 1 and those from three Chinese ginseng collections, Damaya (KC686331), Ermaya (KC686332), and Gaolishen (KC686333) which were retrieved from GenBank. The three Chinese ginseng collections were identical to each other and also to the Korean landrace ‘Jakyung’. By contrast, ‘Sunhyang’ was the most divergent and had the most unique polymorphisms among all accessions (Table 2, S1 Fig).

**Table 2:** Summary of nucleotide polymorphisms in cp genomes and 45S nrDNA sequences of 14 *P*. *ginseng* accessions.

Genome	Type	Position	Nucleotide position^a^	ChP	YP	GU	GO	SO	SU	SP	SH	CS	JK	HS	China
**CP**	SNP	*rps16-trnUUG*	7,159	G	G	G	G	G	G	G	**T**	G	G	G	G
	SNP	*rpoC2* ^b^	21,344	C	C	**T**	C	C	C	C	C	**T**	C	C	C
	SNP	*rpoC1* ^c^	22,287	**T**	G	G	G	G	G	G	G	G	G	G	G
	SNP	*ndhF-rpl32*	115,594	G	G	G	G	G	G	G	**T**	G	G	G	G
	SNP	*ccsA* ^c^	117,376	A	A	**G**	A	A	A	A	A	**G**	A	A	A
	SNP	*ycf1* ^c^	127,069	A	A	A	A	A	A	A	A	A	A	**T**	A
	InDel	*rps16 intron*	5,473	(C)_8_	(C)_8_	**(C)**_**9**_	(C)_8_	(C)_8_	(C)_8_	(C)_8_	(C)_8_	**(C)**_**9**_	(C)_8_	(C)_8_	(C)_8_
	InDel	*rps16-trnUUG*	7,189	13x1	13x1	13x1	13x1	13x1	13x1	13x1	**13x2**	13x1	13x1	13x1	13x1
	InDel	*trnUUC-trnGGU*	32,850								**59**				
	InDel	*trnUGC intron*	105,431/136,936	(G)_11_	(G)_11_	(G)_11_	(G)_11_	(G)_11_	(G)_11_	(G)_11_	**(G)**_**10**_	(G)_11_	(G)_11_	(G)_11_	(G)_11_
	InDel	*ycf1*	111,303/130,896	**57x3**	57x4	57x4	57x4	57x4	57x4	57x4	57x4	57x4	57x4	**57x3**	57x4
	InDel	*rpl32-trnUAG*	115,833	**7x3**	7x2	7x2	7x2	7x2	7x2	7x2	7x2	7x2	7x2	7x2	7x2
**45S nrDNA**	SNP	5.8S rRNA	2,044	A	A	**G**	**G**	A	A	A	A	A	A	A	n/a
	SNP	26S rRNA	4,165	C	C	**G/C** ^**d**^	**G/C** ^**d**^	C	C	C	C	C	C	C	n/a
	SNP	IGS	6,674	A	A	**G**	**G**	A	A	A	A	A	A	A	n/a
	SNP	IGS	7,668	T	T	**C**	**C**	T	T	T	T	T	T	T	n/a
	SNP	IGS	8,365	G	G	G	G	G	G	**T**	G	G	G	G	n/a

Cultivar names: ChP (Chunpoong, KM088019), YP (Yunpoong, KM088020), and others as denoted in Table 1. China indicates identical cp genome sequences of three Chinese collections: P. ginseng isolate Damaya (KC686331), P. ginseng isolate Ermaya (KC686332), P. ginseng isolate Gaolishen (KC686333)

^a^ Nucleotide position is based on cp genome sequence of cultivar Chunpoong and 45S nrDNA sequence of cultivar Chunpoong (KM088019) contig. cp sequence

^b,c^ Non-synonymous and synonymous substitutions, respectively.

^d^ Co-existing heterogeneous nucleotides at the same position.

n/a indicates no available sequence.

We identified six SNPs and six InDels among cp genomes of 14 *P*. *ginseng* cultivars (Fig 2, Table 2). Two SNPs were identified in intergenic spaces and four SNPs in coding sequences. Among the four SNPs in coding regions, three showed non-synonymous substitutions that modify amino acid residues: encoding a glycine (G) vs. serine (S) in *rpoC2*; glutamine (Q) vs. arginine (R) in *ccsA*; and isoleucine (I) vs. asparagine (N) in *ycf1*. Among the six InDels, three are derived from simple insertion or deletion of 1~59 bp nucleotides in a single specific cultivar and the other three originate from copy number variation of tandem repeats ranging from 7 to 57 bp (Table 2).

### Sequence divergence of 45S nrDNAs within *P*. *ginseng* species

**Fig 3:**
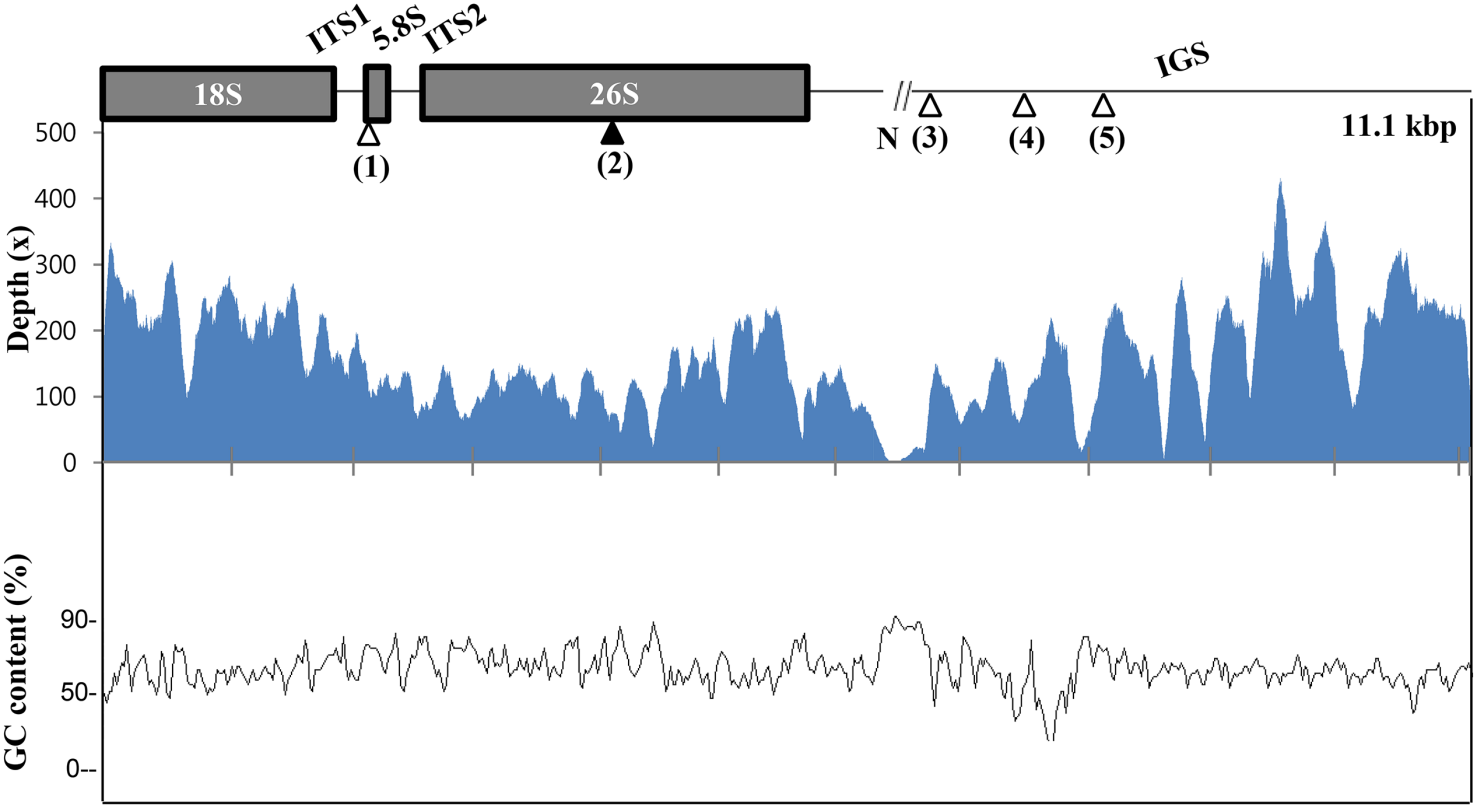
Assembly of complete 45S nrDNA unit. Schematic diagram of a representative complete 45S nrDNA. N indicates gap present in some IGS sequence of nine 45S nrDNAs except Chunpoong and Yunpoong. Polymorphic SNP sites are denoted as arrowheads. (1), (3), (4), and (5) are polymorphic sites among 11 *P*. *ginseng* cultivars and (2) is a heterogeneous site with co-appearance of G and C in *P*. *ginseng* cv. Gumpoong and Gopoong. Mapping of Gumpoong cultivar 11,067 bp raw reads on the 45S nrDNA sequence of Gumpoong. (Accession no. KM207667) GC contents for each region of 45S nrDNA sequence. GC contents were calculated from the GC composition in 100-bp sliding windows.

The 45S nrDNA unit sequences were highly homogeneous among the 11 Korean *P*. *ginseng* cultivars sequenced in this study (Table 1) with 10,095~11,091 bp single units (GenBank accession numbers for the 45S nrDNA of the cultivars are displayed in Table 1). We did not include the three Chinese collections in this analysis because of the lack of reported 45S nrDNA sequences. Some cultivars have a nucleotide gap in an IGS region that has high GC composition and lower WGS read depth (Fig 3). Comparison of 45S nrDNA sequences revealed five SNPs, one in the 5.8S rRNA region, three in IGS sequence, and one in 26S rRNA coding sequence that was heterogeneous, with co-appearance of G and C in cultivars Gumpoong and Gopoong (Table 2). Three SNPs were identified in both Gumpoong and Gopoong, and one SNP was unique to cultivar Sunpoong (Table 2). Large repeat sequences with 3.5 copies of 641-bp sub-repeat elements were identified in IGS regions of all 11 accessions.

### Validation of intra-species polymorphism and development of cultivar authentication markers

To validate the intra-species SNP and InDel polymorphism identified from comparison of complete cp genome sequences and 45S nrDNAs derived from 11 cultivars (nine registered inbred cultivars in Korea, and two Korean local landraces) and also to explore their utility as molecular markers for authentication of ginseng cultivars, we conducted PCR analysis using specific primers targeting the polymorphic sites (Table 3). We inspected four InDel regions and excluded two InDel regions that showed only one bp mono-polymer length difference. We also inspected two SNP regions by designing dCAPS markers. The four InDel markers were newly identified in this study.

**Table 3:** Primers to detect polymorphism among *P*. *ginseng* accessionsPrimer ID.

		Primer sequence (5’- 3’)	Product size (bp)	Location
SNP based dCAPS	pgcpd01^a^	F: AAATATGACCAACAGTAGTTCGAATCTA	212/190	*rpoC1*
		R: AGCTTATCGGCAGAAACGAA		
	pgcpd02^b^	F: ATTTCGGGGACTCACAGAAGTAC	200/177	*rpoC2*
		R: AAAGCAATTTACGCGAAGGA		
InDel based markers	pgcp139f*r2	F: TGTGCGACAAACAAATAAGTCA	157/150	*rpl32 ~ trnUAG*
		R2: CGAAGCGAGTTCCATTTCAT		
	pgycf1	F: GGTATTAGTCTGGATACGGCAAA	729/672/615^c^	*ycf1*
		R: TCGAAAAGAAGGGTCACAAGA		
	pgcp097f2*r	F: TGGAAAGGCTGTTGTCACTG	390/377/344	*rps16 ~ trnUUG*
		R: TCAGCAACGGGAGATATTCA		
	pgcp137	F: TCCTGAACCACTAGACGATGG	514/455	*trnUUC ~ trnGGU*
		R: TTTCGATAACTTCTTGATCCCTCT		

^a, b^ pdcpd01 and pgcpd02 are dCAPS primer pairs with *Xba*I and *Sca*I restriction sites, respectively.

^c^ PCR product size is derived from *P*. *quinquefolius*.

**Fig 4:**
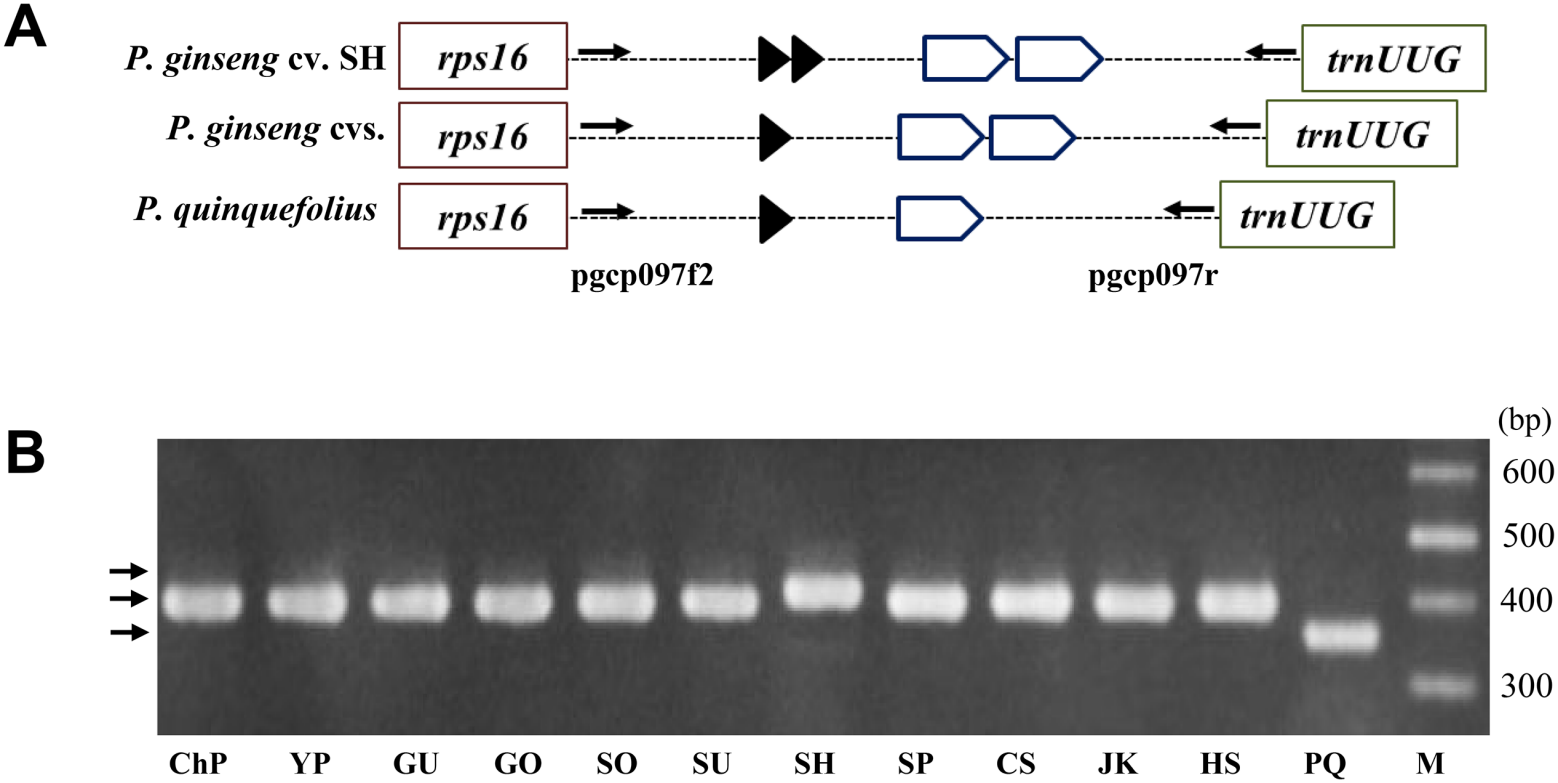
Validation of copy number variation (CNV) of TRs in *rps16 ~ trnUUG* region. (A) Schematic diagram of CNV of TR units among 11 *P*. *ginseng* cultivars and *P*. *quinquefolius*. Arrowheads and polygons indicate TR units of 13 bp and 33 bp, respectively. *P*. *ginseng* cv. SH and *P*. *ginseng* other cvs. indicate cultivar Sunhyang and the remaining 11 accessions listed in Table 1 and *P*. *quinquefolius*. (B) PCR analysis of CNV regions using 097f2*r primer set in 11 *P*. *ginseng* cultivars and *P*. *quinquefolius*. Abbreviated cultivar names (defined in Table 1) are denoted on the gel. PQ and M denote *P*. *quinquefolius* and 100-bp DNA ladder, respectively.

**Fig 5:**
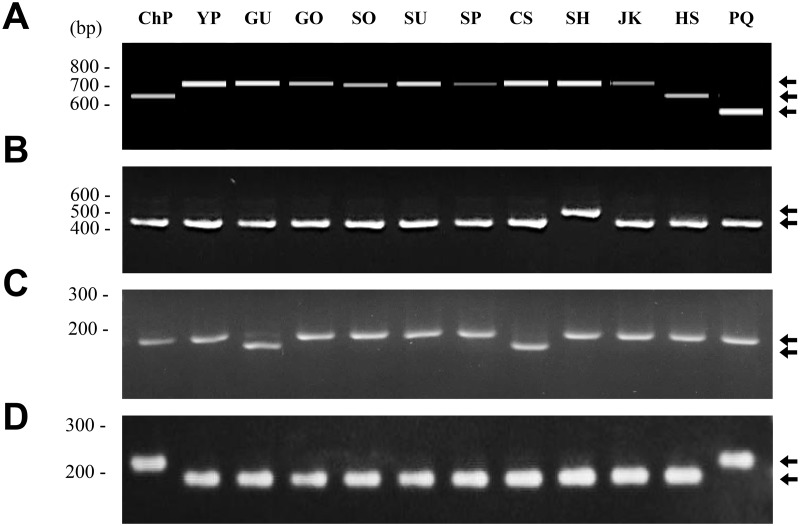
Validation of SNP and InDel polymorphic sites. (A, B) PCR analysis of InDel regions in *ycf1* (A) using primer set pgycf1 and *trnUUC-trnGGU* (B) using primer set pgcp137 (Table 3). (C) SNP analysis using dCAPS primers, pgcpd02, designed for the SNP site in the *rpoC2* gene (Table 3). *Sca*I digestion of the amplicon produced a digested fragment except in GU and CS. (D) SNP analysis with dCAPS primers, pgcpd01, designed for the SNP site in *rpoC1* gene (Table 3). *Xba*I digestion of the amplicon produced a digested fragment except in ChP and PQ. Abbreviated cultivar names (defined in Table 1) are denoted on the gels. PQ and M denote *P*. *quinquefolius* and 100-bp DNA ladder, respectively.

We identified 118 tandem repeats (TRs) (6-57bp) in cp genome sequences of 11 *P*. *ginseng* cultivars. Copy number variation of various TRs played major role in InDel polymorphism. Three of four cultivar-unique polymorphic InDel regions were derived from copy number variations among cultivars. One InDel at intergenic regions of *rps16-trnUUG* derived from copy number variance of two kinds of TRs, 13 bp and 33 bp TRs, was identified in one Korean inbred cultivar ‘Sunhyang’ and *P*. *quinquefolius*. PCR analysis for the target with pgcp097f2*r showed the expected band size differences, with unique bands in ginseng cultivar Sunhyang and *P*. *quinquifolius* (Fig 4A and 4B). A 13-bp TR-based InDel marker, 139f*r2, derived from *rpl32-trnUAG* clearly distinguished Chunpoong from other ginseng cultivars (Tables 2 and 3). The 57-bp TR-based InDel marker pgycf1f*r derived from the *ycf1* gene clearly distinguished Chunpoong and Hwangsook from other *P*. *ginseng* Korean cultivars as well as *P*. *quinquefolius* (Fig 5A). Meanwhile, one 59-bp unique inserted sequence was identified at *trnUUC-trnGGU* in cultivar Sunhyang among all 14 accessions (Fig 5B).

**Fig 6:**
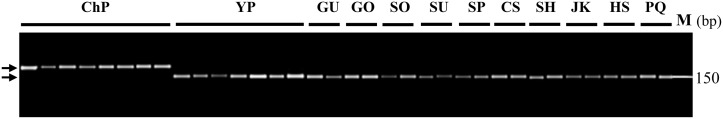
Application of ChP-specific marker, pgcp139f*r2, to individuals of each ginseng cultivar. A total of 72 individuals from 11 *P*. *ginseng* cultivars and *P*. *quinquefolius* (5~20 individuals for each cultivar) were analyzed using PCR marker pgcp139f*r2 and represented eight individuals for ChP and two to seven for the other cultivars. Amplified DNA fragments were separated by capillary electrophoresis using a Fragment analyzer. Abbreviated cultivar names (defined in Table 1) are given above the lanes. PQ and M indicate *P*. *quinquefolius* and DNA size marker, respectively.

We could differentiate some of SNPs using high resolution melting analysis. However, we also designed dCAPS markers for the SNP regions if any restriction enzyme sites were available for the clear validation of the SNP genotype. One SNP in *rpoC2* was unique to Gumpoong and Cheongsun among the other cultivars, and the SNP was detectable using dCAPS markers (Fig 5C). One SNP found in the *rpoC1* exon region was detectable by dCAPS marker and revealed as unique in the cp genome of Chunpoong (Table 2, Fig 5D). We inspected 3~20 individuals for each cultivar and most cultivar-unique bands showed the same genotype for individuals in same cultivar (Fig 6, S2–S5 Figs), indicating that the markers were valuable for cultivar authentication.

## Discussion

### Complete cp genome and nrDNA sequences derived from low-coverage whole-genome NGS data

The application of low coverage NGS data for genome-wide SNP genotyping is usually based on the use of reference genome sequence [22,23]. However, those studies generally do not focus on cp genomes and nrDNA because of their repetitive nature. Here we used low coverage NGS data to obtain complete cp genomes and nrDNA sequences of various *P*. *ginseng* accessions based on *de novo* assembly using low coverage WGS. We successfully obtained the complete sequences of cp and nrDNAs from 11 ginseng cultivars using 0.1X ~ 0.3X low coverage NGS reads. The initial contig numbers for the 11 cultivars varied from 2 ~ 6 to cover the complete cp genome. However, the breakpoints were common among different cultivars, indicating that our assembly method can be efficiently utilized for obtaining the complete cp genome from large numbers of samples.

### SNPs and InDels at the inter- and intra-species level for *Panax*

The chloroplast genome and nrDNA are highly conserved within species, and nucleotide substitutions in those sequences have been used to examine plant evolution and genome differentiation between species [11,24]. Therefore, cp genome and nrDNA are useful targets for DNA barcoding to authenticate taxon. Within the cp genome, the *matK* and *rbcL* genes are main barcoding sites for land plants [25]. In addition, some coding regions including *rpoC2*, *rpoC1*, *ycf1* have been identified as hotspot regions for variation [26,27]. We also investigated sequence variation between *P*. *ginseng* and its most closely related species, *P*. *quinquefolius* (Accession no. KM088018) [11]. We identified 137 SNPs and 39 InDels from complete cp genome sequences and eight SNPs and two InDels from 45S nrDNA. Eight of the 11 core-barcoding sites described previously [25] were also polymorphic between the two *Panax* species.

Although intra-species polymorphism is rare in the barcoding sites, we were able to detect some valuable and unique polymorphic markers to authenticate ginseng cultivars. We identified six SNPs and six InDels in the complete cp genome sequences of ginseng at the intra-species level. Among the 12, seven were derived from genic regions and five were in intergenic regions. We also found five SNPs at two genic and three intergenic regions in the complete 45S nrDNAs. The three Chinese *P*. *ginseng* collections did not show unique differences compared to Korean accessions, indicating that the 12 polymorphic sites represent most of the intra-species polymorphism in *P*. *ginseng*.

### Hotspot polymorphic sites in the cp genome of *Panax* species

Copy number variation of TRs distributed in cp genomes is the main source of diversity at the intra- and inter species level. The largest amount of variation was in the *ycf1* gene, reflecting copy number variation of a 57-bp TR among *P*. *ginseng* cultivars as well as the related species *P*. *quinquefolius* (Fig 5A). Chunpoong and Hwangsook had three copies, while the remaining seven cultivars of *P*. *ginseng* had four copies and *P*. *quinquefolius* had two copies. Overall, the copy number variation of this 57-bp TR is a major contributor to the variation in cp genome size among *P*. *ginseng* cultivars and *Panax* species.

A second region of diversity was found in the intergenic spacer between *rp132* and *trnUAG*. In particular, cultivar Chunpoong had three copies of a 7-bp TR (ACCTATT), while other *Panax* accessions had two copies of it (Table 2). The polymorphism derived from copy numbers of 7-bp TR was unique only to Chunpoong among all the *P*. *ginseng* cultivars and *P*. *quinquifolius* individuals (Fig 6), which indicates that this is a valuable authentication marker for the cultivar Chunpoong. The third highly polymorphic area was in *rps16-trnUUG*, which showed intra- and inter-species polymorphism based on copy number variation of two different TRs among *P*. *ginseng* cultivars and with *P*. *quinquefolius*. Sunhyang had two copies of a 13-bp TR unit, while the others including *P*. *quinquefolius* had only one copy of that TR. Copy number variation of a 33-bp TR was detected between *P*. *ginseng* and *P*. *quinquefolius* (Fig 4A).

### Development of molecular markers for authentication of ginseng cultivars

Cp-genome derived markers are convenient and reliable for authentication of plant species because the cp DNA is high copy and resistant to mechanical breakdown due to its small and stable circular form compared to nuclear DNA. In *P*. *ginseng*, the recently duplicated allotetraploid nuclear genome structure makes it difficult to detect polymorphic SSR markers derived from the nuclear genome [11,28,29]. In this study, we were able to develop cp-derived cultivar-specific markers for three ginseng cultivars, Chunpoong, Sunhyang and Hwangsook. Sunhyang has the most abundant polymorphism in the cp genome; we identified five Sunhyang-unique markers, comprising two SNPs and three InDels. Chunpoong has two cultivar-unique markers, one SNP and one InDel, which could be easily identified from other cultivars and individual plants (Figs 5A and 6). Hwangsook has one cultivar-specific SNP (Table 2), and Sunpoong has one cultivar-specific SNP derived from 45S nrDNA. Gumpoong, Gopoong, and Cheongsun can be differentiated from each other based on a combination of polymorphic sites derived from cp genome and nrDNA, although Gumpoong and Cheongsun have identical cp genomes. Overall, whereas Yunpoong, Sunun, Sunone and Jakyung were identical for both cp genomes and 45S nrDNAs (Table 2), the other eight cultivars can be authenticated using one or a few marker combinations in 17 polymorphic sites. These markers will be valuable to authenticate cultivars using fresh tissues or even with processed root products. We previously described a six SSR marker-derived authentication system for nine registered ginseng cultivars; Yunpoong, Sunone and Sunun could be authenticated using these nuclear SSR markers [28,30] as a backup for authentication based on the cp-derived markers developed in this study.

Taken together, we report 17 high-value polymorphic sites showing intra-species level sequence variation in the cp genome and nrDNA of *P*. *ginseng* (Table 2). The polymorphisms found in this study can be used to elucidate evolutionary history such as the origin of *Panax* species or accessions at the inter- and intra-species level. Furthermore, the polymorphic sites promote practical applications for molecular analysis to protect ginseng cultivars and the ginseng industry. Breeding new cultivars takes a long time due to the long life-cycle of *P*. *ginseng* [2]. These markers will contribute to maintain the purity of each cultivar by protection against unintentional contamination and thus promote the high-value ginseng industry.

## Supporting Information

S1 Fig Comparison of cp genome sequences of *P*. *ginseng* cultivars. Complete cp genomes of 11 *P*. *ginseng* cultivars generated in this study and cp genomes of *P*. *ginseng* (Accession no. NC_006290) and *P*. *quinquefolius* (Accession no. KM088018) were used for comparison. Genic regions were identified using the DOGMA program (http://dogma.ccbb.utexas.edu/) and the comparative map was prepared using mVISTA (http://genome.lbl.gov/vista/mvista/submit.shtml). Blue block, conserved gene; Sky-blue block, tRNA and rRNA; Red block, intergenic region. The *ycf1* gene region was identified as a hot spot for chloroplast sequence divergence.
(DOCX)

S2 Fig Classification of individuals of 12 cultivars based on SNP in the *rpoC1* gene. The SNP site was analyzed by dCAPS primers, pgcpd01, designed for the SNP site in *rpoC1* (Table 3). More than three individual plants for each *P*. *ginseng* cultivar were analyzed and *Xba*I digestion revealed unique patterns for ChP and PQ plants. Size difference of fragments was confirmed by 3% agarose gel electrophoresis **(A)** and by capillary electrophoresis using a Fragment analyzer **(B)**. Red arrowheads indicate undigested fragments in ChP and PQ plants. Abbreviated cultivar names (defined in Table 1) are above the gels. PQ and M denote *P*. *quinquefolius* and DNA size markers, respectively.
(DOCX)

S3 Fig Classification of individuals of 12 cultivars based on InDel regions in *trnUUC-trnGGU*. Three individual plants were analyzed for each cultivar using primer set pgcp137 (Table 3). Red arrowhead indicates amplicons in SH, which were longer by a 59-bp insertion in the *trnUUC-trnGGU* region compared to the other cultivars. Abbreviated cultivar names (defined in Table 1) are above the gels. PQ and M denote *P*. *quinquefolius* and DNA size markers, respectively.
(DOCX)

S4 Fig Classification of individuals of 12 cultivars based on InDel regions in *ycf1* gene. More than nine plants were analyzed for each cultivar using primer set pgycf1 (Table 3). Red arrowheads indicate amplicons in ChP (C) and HS (H), which were smaller by a 57-bp deletion in the *ycf1* gene compared to the other cultivars. O denotes cultivars other than ChP and HS. M indicates DNA size markers.
(DOCX)

S5 Fig Classification of cultivars based on SNP in the *rpoC2* gene. dCAPS primers, pgcpd02, designed for the SNP site in the *rpoC2* gene (Table 3) were applied to more than three individual plants of each *P*. *ginseng* cultivar. Red arrowhead indicates *Sca*I-digested fragments in GU and GS plants.
(DOCX)
