# Supplementary material for: Comprehensive Survey of Genetic Diversity in Chloroplast Genomes and 45S nrDNAs within Panax ginseng Species
Source: PLoS One. 2015 Jun 10;10(6):e0117159. doi: 10.1371/journal.pone.0117159 (PMC4465672; doi:10.1371/journal.pone.0117159)
Supplement: S1 Fig — Complete cp genomes of 11 P. ginseng cultivars generated in this study and cp genomes of P. ginseng (Accession no. NC_006290) and P. quinquefolius (Accession no. KM088018) were used for comparison. Genic regions were identified using the DOGMA program (http://dogma.ccbb.utexas.edu/) and the comparative map was prepared using mVISTA (http://genome.lbl.gov/vista/mvista/submit.shtml). Blue block, conserved gene; Sky-blue block, tRNA and rRNA; Red block, intergenic region. The ycf1 gene region was identified as a hot spot for chloroplast sequence divergence. (DOCX) [file pone.0117159.s001.docx]

**Supporting Information**


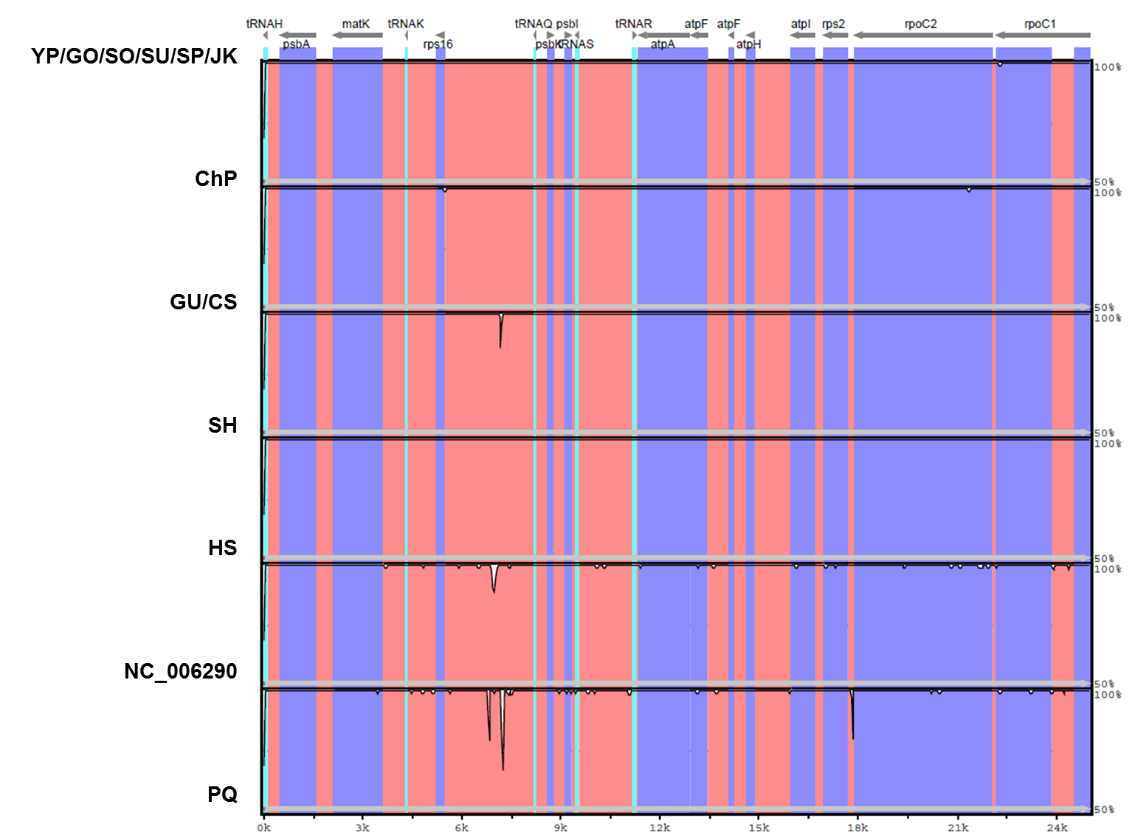


**
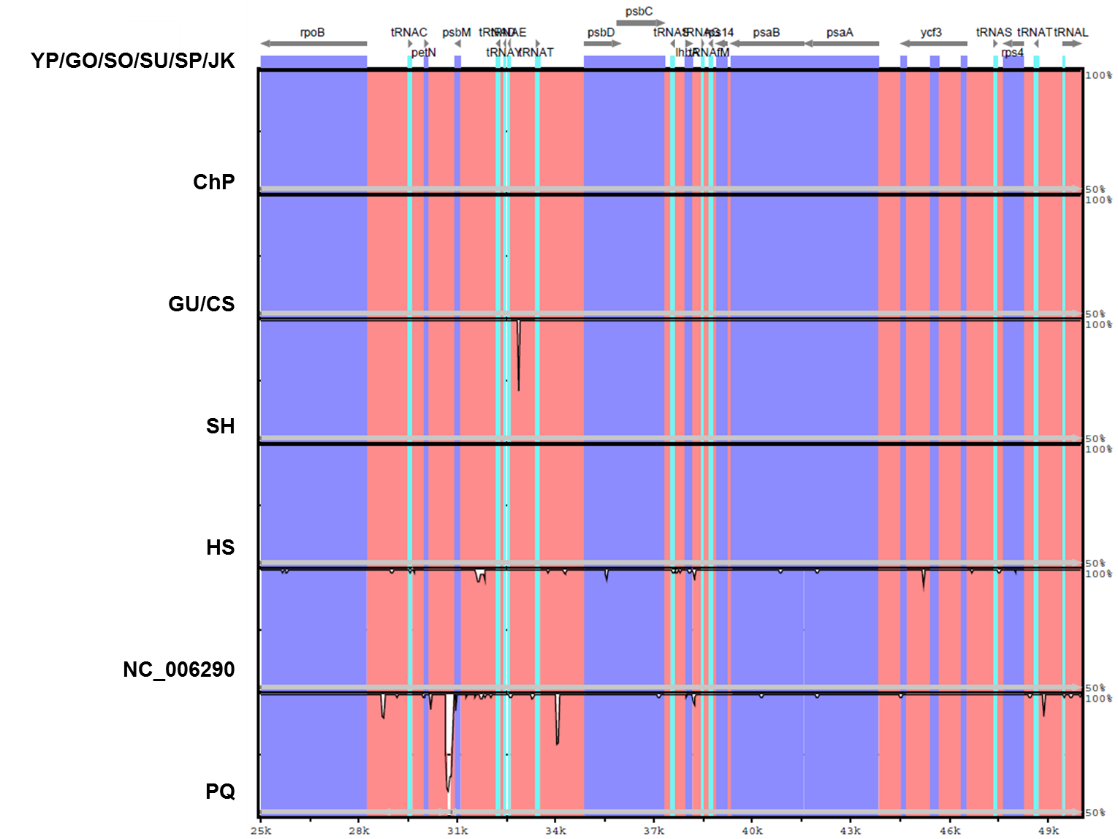
**

**
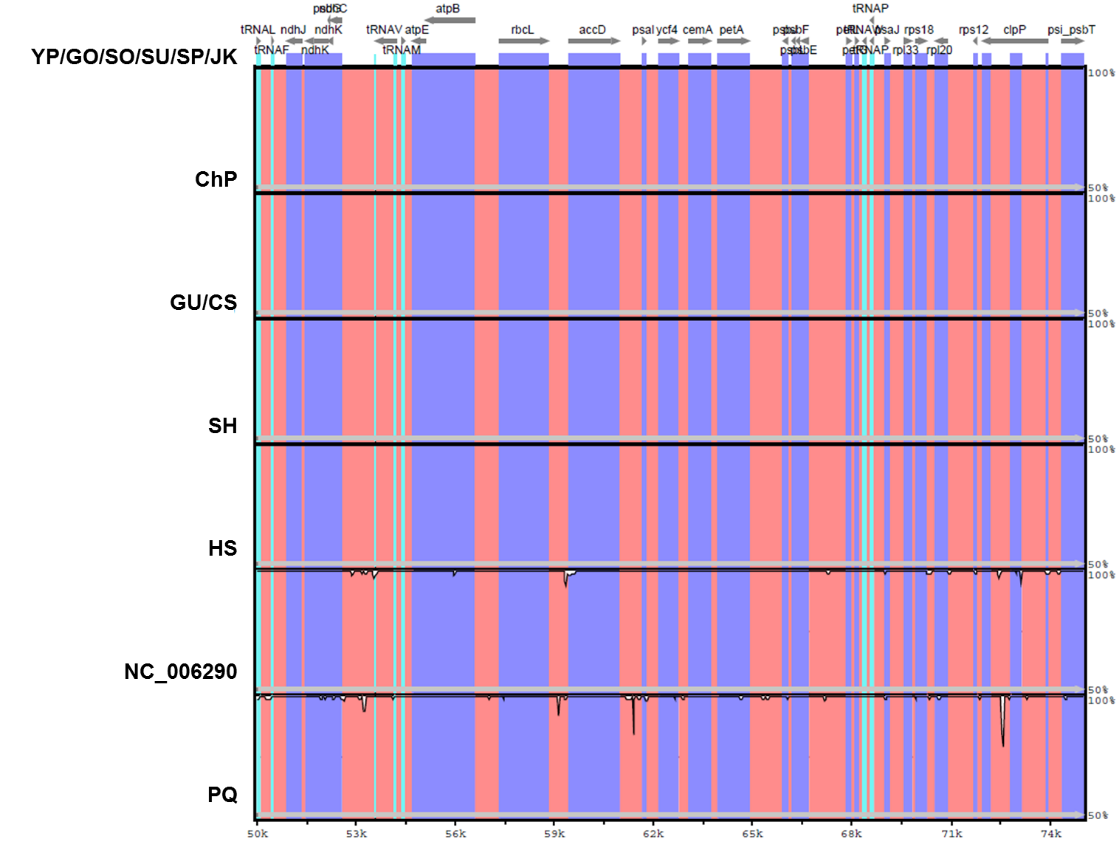
**

**
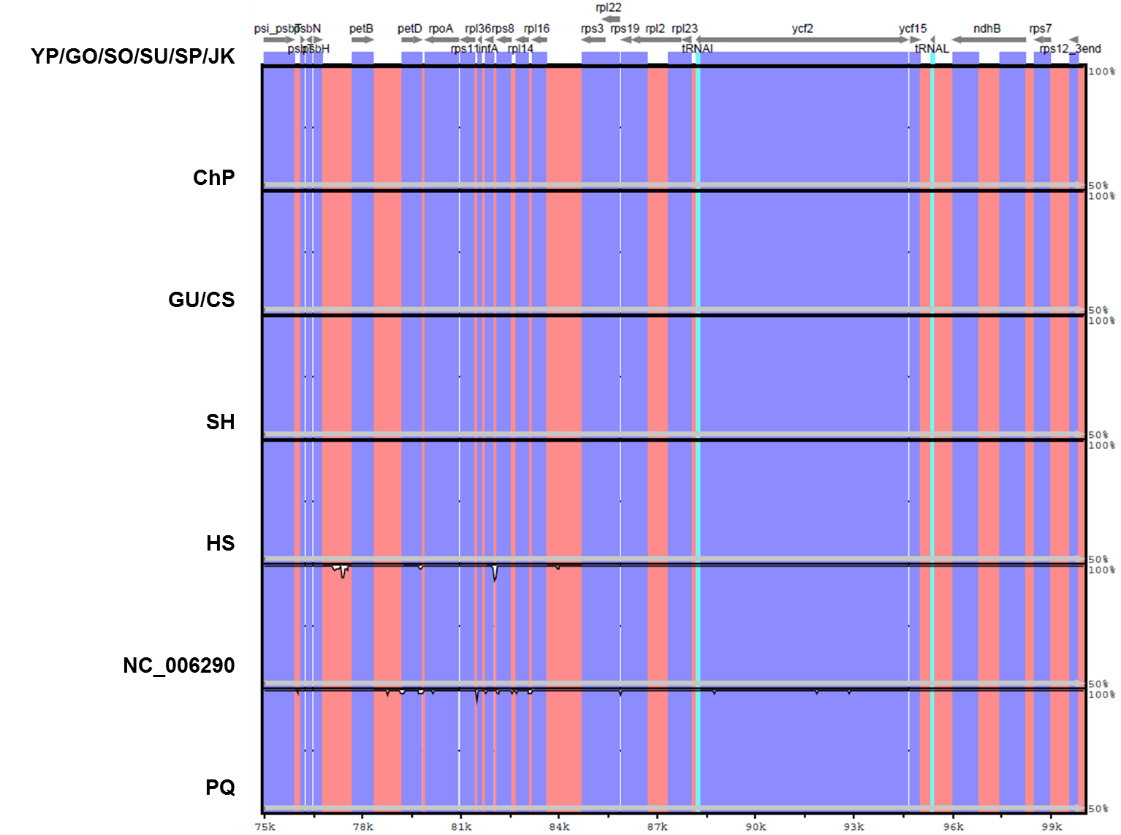
**


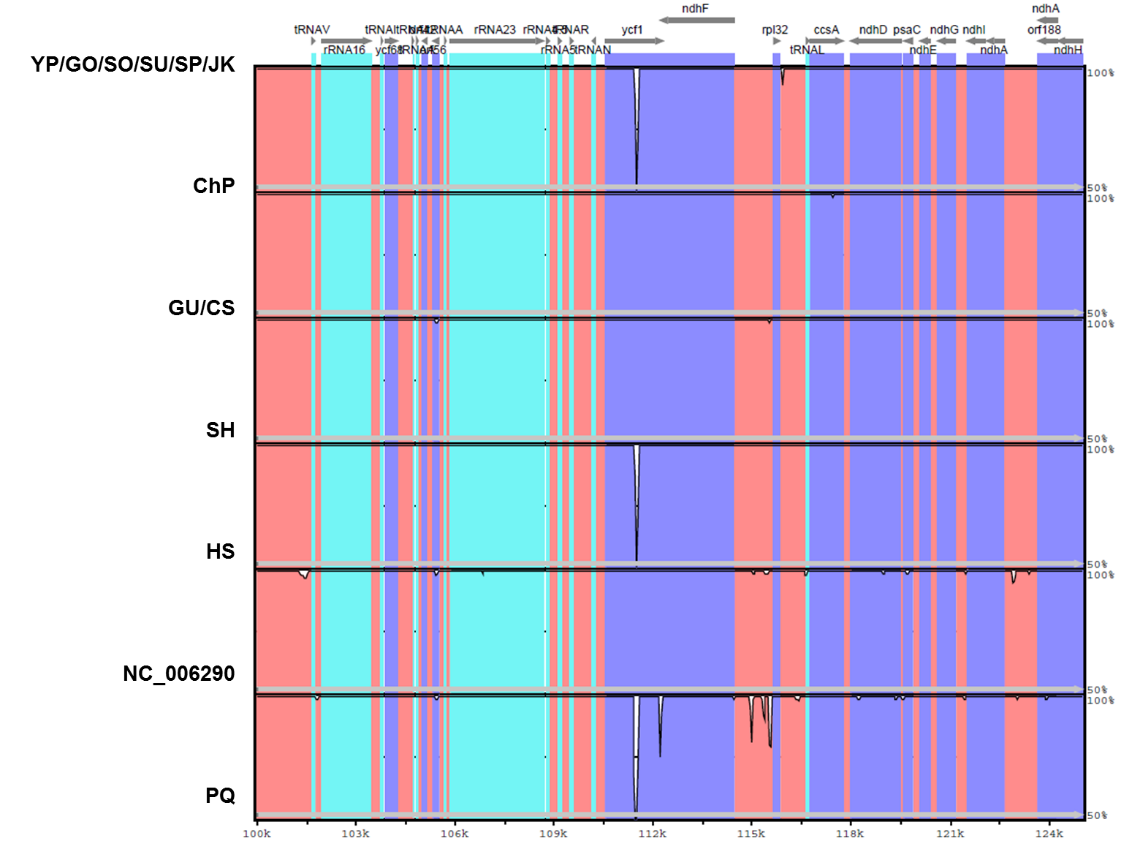


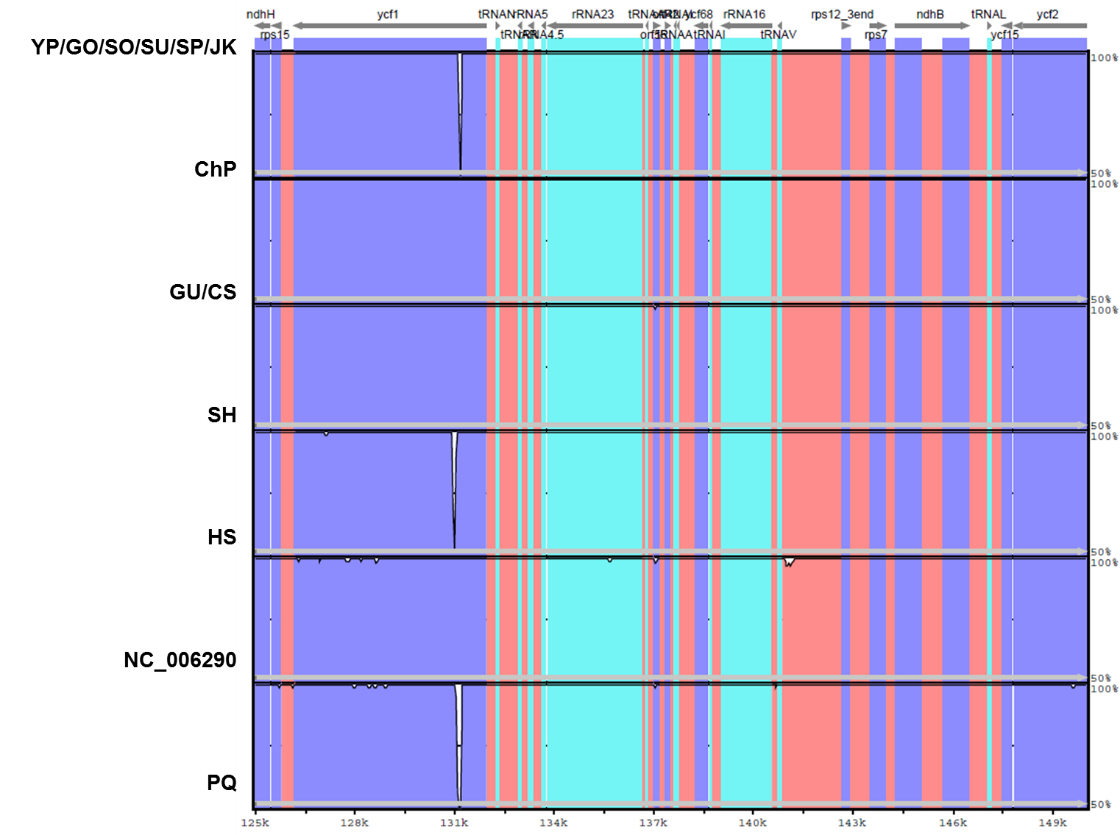


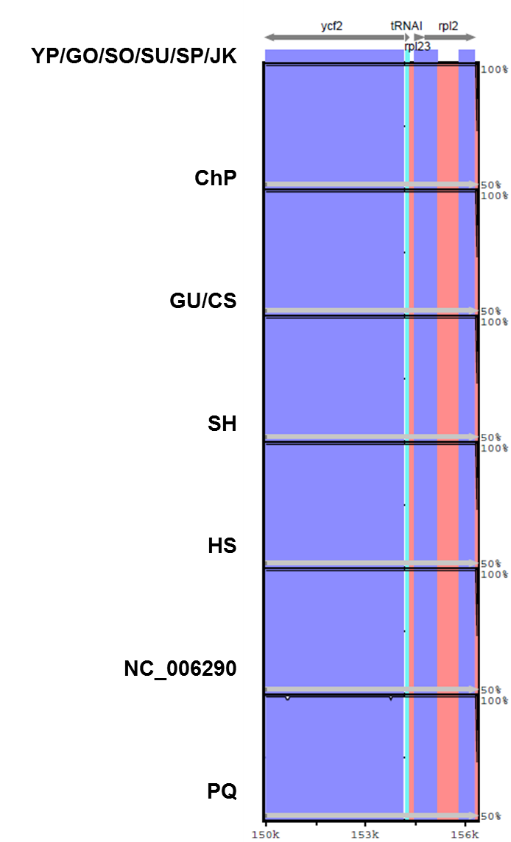


**Figure S1**. Comparison of cp genome sequences of *P. ginseng* cultivars. Complete cp genomes of nine *P.* *ginseng* cultivars generated in this study and previously reported cp genomes of cv. Chunpoong (Accession no. KM088019), cv. Yunpoong (Accession no. KM088020), and *P. quinquefolius* (Accession no. KM088018) were used for comparison. Genic regions were identified using the DOGMA program (<http://dogma.ccbb.utexas.edu/>) and the comparative map was prepared using mVISTA (<http://genome.lbl.gov/vista/mvista/submit.shtml>). Blue block, conserved gene; Sky-blue block, tRNA and rRNA; Red block, intergenic region. The *ycf1* gene region was identified as a hot spot for chloroplast sequence divergence.
