# Supplementary material for: Comprehensive Survey of Genetic Diversity in Chloroplast Genomes and 45S nrDNAs within Panax ginseng Species
Source: PLoS One. 2015 Jun 10;10(6):e0117159. doi: 10.1371/journal.pone.0117159 (PMC4465672; doi:10.1371/journal.pone.0117159)
Supplement: S2 Fig — The SNP site was analyzed by dCAPS primers, pgcpd01, designed for the SNP site in rpoC1 (Table 3). More than three individual plants for each P. ginseng cultivar were analyzed and XbaI digestion revealed unique patterns for ChP and PQ plants. Size difference of fragments was confirmed by 3% agarose gel electrophoresis (A) and by capillary electrophoresis using a Fragment analyzer (B). Red arrowheads indicate undigested fragments in ChP and PQ plants. Abbreviated cultivar names (defined in Table 1) are above the gels. PQ and M denote P. quinquefolius and DNA size markers, respectively. (DOCX) [file pone.0117159.s002.docx]

**Supporting Information**

**Figure S2**. Classification of individuals of 12 cultivars based on SNP in the *rpoC1* gene. The SNP site was analyzed by dCAPS primers, pgcpd01, designed for the SNP site in *rpoC1* (Table 3). More than three individual plants for each *P. ginseng* cultivar were analyzed and *Xba*I digestion revealed unique patterns for ChP and PQ plants. Size difference of fragments was confirmed by 3% agarose gel electrophoresis **(A)** and by capillary electrophoresis using a Fragment analyzer **(B)**. Red arrowheads indicate undigested fragments in ChP and PQ plants. Abbreviated cultivar names (defined in Table 1) are above the gels. PQ and M denote *P. quinquefolius* and DNA size markers, respectively.
