# Supplementary material for: Comprehensive Survey of Genetic Diversity in Chloroplast Genomes and 45S nrDNAs within Panax ginseng Species
Source: PLoS One. 2015 Jun 10;10(6):e0117159. doi: 10.1371/journal.pone.0117159 (PMC4465672; doi:10.1371/journal.pone.0117159)
Supplement: S3 Fig — Three individual plants were analyzed for each cultivar using primer set pgcp137 (Table 3). Red arrowhead indicates amplicons in SH, which were longer by a 59-bp insertion in the trnUUC-trnGGU region compared to the other cultivars. Abbreviated cultivar names (defined in Table 1) are above the gels. PQ and M denote P. quinquefolius and DNA size markers, respectively. (DOCX) [file pone.0117159.s003.docx]

**Supporting Information**

**Figure S3**. Classification of individuals of 12 cultivars based on InDel regions in *trnUUC-trnGGU.* Three individual plants were analyzed for each cultivar using primer set pgcp137 (Table 3). Red arrowhead indicates amplicons in SH, which were longer by a 59-bp insertion in the *trnUUC-trnGGU* region compared to the other cultivars. Abbreviated cultivar names (defined in Table 1) are above the gels. PQ and M denote *P. quinquefolius* and DNA size markers, respectively.
