# Supplementary material for: Comprehensive Survey of Genetic Diversity in Chloroplast Genomes and 45S nrDNAs within Panax ginseng Species
Source: PLoS One. 2015 Jun 10;10(6):e0117159. doi: 10.1371/journal.pone.0117159 (PMC4465672; doi:10.1371/journal.pone.0117159)
Supplement: S4 Fig — More than nine plants were analyzed for each cultivar using primer set pgycf1 (Table 3). Red arrowheads indicate amplicons in ChP (C) and HS (H), which were smaller by a 57-bp deletion in the ycf1 gene compared to the other cultivars. O denotes cultivars other than ChP and HS. M indicates DNA size markers. (DOCX) [file pone.0117159.s004.docx]

**Supporting Information**

**Figure S4**. Classification of individuals of 12 cultivars based on InDel regions in *ycf1* gene. More than nine plants were analyzed for each cultivar using primer set pgycf1 (Table 3). Red arrowheads indicate amplicons in ChP (C) and HS (H), which were smaller by a 57-bp deletion in the *ycf1* gene compared to the other cultivars. O denotes cultivars other than ChP and HS. M indicates DNA size markers.
