# Supplementary material for: Comprehensive Survey of Genetic Diversity in Chloroplast Genomes and 45S nrDNAs within Panax ginseng Species
Source: PLoS One. 2015 Jun 10;10(6):e0117159. doi: 10.1371/journal.pone.0117159 (PMC4465672; doi:10.1371/journal.pone.0117159)
Supplement: S5 Fig — dCAPS primers, pgcpd02, designed for the SNP site in the rpoC2 gene (Table 3) were applied to more than three individual plants of each P. ginseng cultivar. Red arrowhead indicates ScaI-digested fragments in GU and GS plants. (DOCX) [file pone.0117159.s005.docx]

**Supporting Information**

**Figure. S5.** Classification of cultivars based on SNP in the *rpoC2* gene. dCAPS primers, pgcpd02, designed for the SNP site in the *rpoC2* gene (Table 3) were applied to more than three individual plants of each *P. ginseng* cultivar. Red arrowhead indicates *Sca*I-digested fragments in GU and GS plants.
